# Assessing the pharmacokinetic profile of the CamMedNP natural products database: an *in silico* approach

**DOI:** 10.1186/2191-2858-3-10

**Published:** 2013-08-30

**Authors:** Fidele Ntie-Kang, James A Mbah, Lydia L Lifongo, Luc C Owono Owono, Eugene Megnassan, Luc Meva'a Mbaze, Philip N Judson, Wolfgang Sippl, Simon MN Efange

**Affiliations:** 1 CEPAMOQ, Faculty of Science, University of Douala, P.O. Box 8580, Douala, Cameroon; 2 Chemical and Bioactivity Information Centre, Department of Chemistry, Faculty of Science, University of Buea, P.O. Box 63, Buea, Cameroon; 3 Department of Pharmaceutical Sciences, Martin Luther University of Halle-Wittenberg, Wolfgang-Langenbeck Str. 4, Halle (Saale) 06120, Germany; 4 Department of Chemistry, Faculty of Science, University of Buea, P.O. Box 63, Buea, Cameroon; 5 Laboratory for Simulations and Biomolecular Physics, Advanced Teachers Training College, University of Yaoundé I, P.O. Box 47, Yaoundé, Cameroon; 6 Laboratory of Fundamental and Applied Physics, University of Abobo-Adjame, Abidjan 02 BP 801, Cote d'Ivoire, Africa; 7 Department of Chemistry, Faculty of Science, University of Douala, P.O. Box 24157, Douala, Cameroon; 8 Chemical and Bioactivity Information Centre, 22-23 Blenheim Terrace, Woodhouse Lane, Leeds LS2 9HD, UK

**Keywords:** ADMET, Database collection, Descriptors, *In silico*, Medicinal plants, Natural products

## Abstract

**Background:**

Drug metabolism and pharmacokinetic (DMPK) assessment has come to occupy a place of interest during the early stages of drug discovery today. Computer-based methods are slowly gaining ground in this area and are often used as initial tools to eliminate compounds likely to present uninteresting pharmacokinetic profiles and unacceptable levels of toxicity from the list of potential drug candidates, hence cutting down the cost of the discovery of a drug.

**Results:**

In the present study, we present an *in silico* assessment of the DMPK profile of our recently published natural products database of 1,859 unique compounds derived from 224 species of medicinal plants from the Cameroonian forest. In this analysis, we have used 46 computed physico-chemical properties or molecular descriptors to predict the absorption, distribution, metabolism and elimination (ADME) of the compounds. This survey demonstrated that about 50% of the compounds within the Cameroonian medicinal plant and natural products (CamMedNP) database are compliant, having properties which fall within the range of ADME properties of >95% of currently known drugs, while >73% of the compounds have ≤2 violations. Moreover, about 72% of the compounds within the corresponding ‘drug-like’ subset showed compliance.

**Conclusions:**

In addition to the previously verified levels of ‘drug-likeness’ and the diversity and the wide range of measured biological activities, the compounds in the CamMedNP database show interesting DMPK profiles and, hence, could represent an important starting point for hit/lead discovery from medicinal plants in Africa.

## Background

Natural products (NPs) play an increasingly important role in drug discovery today [1-5], both serving as drugs and as templates for the design of nature-inspired medicines [3,6]. In fact, it has been reported that a significant proportion of drugs that undergo clinical trials are either naturally occurring or are derived from NPs [7]. What characterises NPs are their richness in stereogenic centres and coverage of segments of chemical space which are typically not occupied by a majority of synthetic molecules and drugs [8,9]. In addition, they generally contain more oxygen atoms and less aromatic atoms on average, when compared with ‘drug-like’ molecules [8-11]. It is needless to say that NPs sometimes fail the famous ‘drug-likeness’ test due to the often bulky nature of naturally occurring metabolites [11].

It is also worth mentioning that designing drug-like molecules having interesting pharmacokinetic properties is an important paradigm in drug discovery programs [12,13]. This entails the search for lead compounds which can be easily orally absorbed, easily transported to their desired site of action, not easily attacked by metabolising enzymes so as to form toxic metabolic products before reaching the targeted site of action and easily eliminated from the body before accumulating in sufficient amounts that may produce adverse side effects. The ensemble of the above properties is often referred to as absorption, distribution, metabolism and elimination (ADME) properties, or better still ADMET or ADME/T or ADMETox (i.e. if toxicity criteria are also taken into consideration).

Computer-based *in silico* approaches for the prediction of ADMET profiles of drug leads at early stages of drug discovery are increasingly gaining ground [14-16]. This could be explained by the relative cost advantage added to the time factor, when compared to standard experimental approaches for ADMET profiling [17,18]. On these grounds, several theoretical methods for the determination of ADMET parameters have been developed and implemented in a number of currently available software for drug discovery protocols [19-22], even though the predictions are sometimes disappointing [23]. Such software often make use of quantitative structure-activity relationships [22-24] or knowledge-base methods [25-27]. The goal has been to considerably cut down on the currently very high cost of discovery of a drug [17]. A promising lead is often defined as a compound which combines potency with an admirable ADMET profile. As such, compounds with unfavourably predicted pharmacokinetic profiles are either completely dismissed from the list of potential drug candidates (even if they prove to be highly potent) or the drug metabolism and pharmacokinetics (DMPK) properties are ‘fine tuned’ in order to improve their chances of making it to clinical trials [28]. This explains why the ‘graveyard’ of very highly potent compounds which do not make it to clinical trials keeps filling up, to the extent that the process of drug discovery often presents the challenge of either resorting to new leads or ‘resurrecting’ some buried leads with the view of fine-tuning their ADMET profiles.

In a recent paper, we have presented a database of 1,859 compounds derived from the Cameroonian flora, Cameroonian medicinal plant and natural products (CamMedNP), the compounds being predicted to be sufficiently orally available and diverse to be employed in lead discovery programs [29]. Additional arguments in favour of the use of this database are the wide range of the previously observed biological activities of the compounds and the wide range of ailments being treated by traditional medicine with the help of the herbs from which the compounds have been derived [29,30].

Numerous drugs at a late stage of pharmaceutical development and many more lead compounds fail due to adverse pharmacokinetic properties [18]. It is, therefore, important to incorporate the prediction of the ADME properties into the lead compound selection, by means of molecular descriptors. A molecular descriptor is often defined as a structural or physico-chemical property of a molecule or part of a molecule, for example the logarithm of the *n*-octanol/water partition coefficient (log *P*), molar weight (MW) and total polar surface area. A number of relevant molecular properties (descriptors) are often used to help predict the pharmacokinetic behaviour of potential drug leads. In the present study, we have carried out an *in silico* assessment of the ADMET profile of the CamMedNP database by the use of computed molecular descriptors currently implemented in a wide range of software tools as indicators of the pharmacokinetic properties of a large proportion of currently known drugs.

## Methods

### Data sources and generation of 3D structures

The plant sources, geographical collection sites, chemical structures of pure compounds and their measured biological activities were retrieved from literature sources and have been previously described [29]. The three-dimensional (3D) structures were generated using the builder module of MOE [31], and energy minimization was subsequently carried out using the MMFF94 [32] until a gradient of 0.01 kcal/mol was reached.

### Initial treatment of chemical structures and calculation of ADMET-related descriptors

The 1,859 low-energy 3D chemical structures in the CamMedNP library were saved in mol2 format and initially treated with LigPrep [33], distributed by Schrodinger, Inc. (New York, USA). This implementation was carried out with the graphical user interface of the Maestro software package (New York, USA) [34], using the OPLS force field [35-37]. Protonation states at biologically relevant pH were correctly assigned (group I metals in simple salts were disconnected, strong acids were deprotonated and strong bases protonated, while topological duplicates and explicit hydrogens were added). All molecular modelling was carried out on a Linux workstation (San Francisco, USA) with a 3.5 GHz Intel Core2 Duo processor (Santa Clara, USA). A set of the ADMET-related properties (a total of 46 molecular descriptors) were calculated using the QikProp program (New York, USA) [21] running in normal mode. QikProp generates physically relevant descriptors and uses them to perform ADMET predictions. An overall ADME-compliance score, drug-likeness parameter (indicated by #stars), was used to assess the pharmacokinetic profiles of the compounds within the CamMedNP library. The #stars parameter indicates the number of property descriptors computed by QikProp, which falls outside the optimum range of values for 95% of known drugs. The methods implemented were developed by Jorgensen et al. [38-40]. Among the calculated descriptors are the total solvent-accessible molecular surface, *S*_mol_ in Å^2^ (probe radius 1.4 Å; range for 95% of drugs is 300 to 1,000 Å^2^); the hydrophobic portion of the solvent-accessible molecular surface, *S*_mol,hfob_ in Å^2^ (probe radius 1.4 Å; range for 95% of drugs is 0 to 750 Å^2^); the total volume of molecule enclosed by solvent-accessible molecular surface, *V*_mol_ in Å^3^ (probe radius 1.4 Å; range for 95% of drugs is 500 to 2,000 Å^3^); the logarithm of aqueous solubility, log*S*_wat_ (range for 95% of drugs is −6.0 to 0.5) [36,38]; the logarithm of predicted binding constant to human serum albumin, log*K*_HSA_ (range for 95% of drugs is −1.5 to 1.2) [41]; the logarithm of predicted blood/brain barrier partition coefficient, log *B/B* (range for 95% of drugs is −3.0 to 1.0) [42-44]; the predicted apparent Caco-2 cell membrane permeability (BI*P*_Caco-2_) in Boehringer-Ingelheim scale, in nm/s (range for 95% of drugs is <5 low, >100 high) [45-47]; the predicted apparent Madin-Darby canine kidney (MDCK) cell permeability in nm s^−1^ (<25 poor, >500 great) [46]; the index of cohesion interaction in solids, Ind_coh_, calculated from the number of hydrogen bond acceptors (HBA), hydrogen bond donors (HBD) and the surface area accessible to the solvent (*S*_mol_) by the relation $\mathrm{In}d_{\mathrm{coh}}=\mathrm{HBA}\times \sqrt{\mathrm{HBD}}/S_{\mathrm{mol}}$ (0.0 to 0.05 for 95% of drugs) [40]; the globularity descriptor, Glob = (4*πr*^2^)/*S*_mol_, where *r* is the radius of the sphere whose volume is equal to the molecular volume (0.75 to 0.95 for 95% of drugs); the predicted polarizability, QP_polrz_ (13.0 to 70.0 for 95% of drugs); the predicted IC_50_ value for blockage of HERG K^+^ channels, logHERG (concern <−5) [48,49]; the predicted skin permeability, log*K*_p_ (−8.0 to −1.0 for 95% of drugs) [50,51]; and the number of likely metabolic reactions, #metab (range for 95% of drugs is 0 to 15).

## Results and discussion

### Overall DMPK compliance of the CamMedNP library

**Figure 1:**
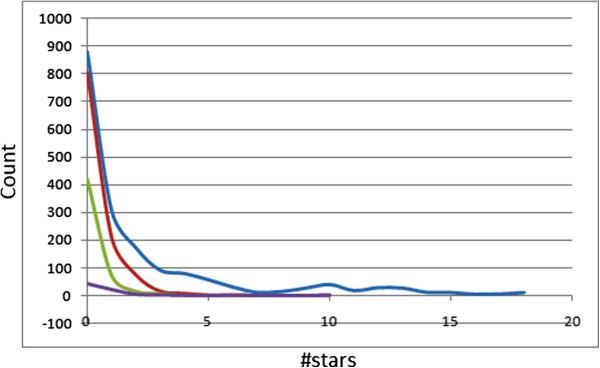
**Distribution curves for #stars within the CamMedNP library and subsets.** Blue = CamMedNP library, red = drug-like subset, green = lead-like subset and violet = fragment-like subset.

The 24 most relevant molecular descriptors calculated by QikProp are used to determine the #star parameter [52]. A plot of the #stars parameter (on the *x*-axis) against the corresponding counts (on the *y*-axis) in the CamMedNP is shown within the same set of axes with those of the ‘drug-like’, ‘lead-like’ and ‘fragment-like’ standard subsets, Figure 1. The criteria for the respective standard subsets were defined as MW < 500, log *P* < 5, HBD ≤ 5, HBA ≤ 10 [14]; 150 ≤ MW ≤ 350, log *P* ≤ 4, HBD ≤ 3, HBA ≤ 6 [53-55] and MW ≤ 250, −2 ≤ log *P* ≤ 3, HBD < 3, HBA < 6, NRB < 3 [56]. QikProp was unable to compute the ADMET descriptors for 25 compounds out of the total library due to limitations that were not clear to us. Of the remaining 1,834 compounds, 48.04% showed #star = 0, while 74.21% had #star ≤ 2. Among the 1,122 compounds of the drug-like subset, 79.12% had pharmacokinetic descriptors within the acceptable range for 95% of known drugs, while 97.33% showed #stars ≤ 2. The lead-like and fragment-like subsets were, respectively, 81.15% and 55.56% compliant for all of the 24 most relevant computed descriptors. The mean values for 19 selected computed descriptors have been shown in Table 1 for all four compound libraries, while the percentage compliances for 14 selected ADMET-related descriptors are shown in Table 2. The mean values and percentage compliances indicate a high probability of finding drug leads within the CamMedNP compound library.

**Table 1:** Average pharmacokinetic property distributions of total CamMedNP library in comparison with various subsets

	**Library name**	**Subset**		
	**CamMedNP**	**Drug-like**	**Lead-like**	**Fragment-like**
Lib. size^a^	1,859	1,122	520	81
No. compl.^b^	881	807	422	45
MW (Da)^c^	426.70	330.33	276.46	195.75
Log *P*^d^	4.18	2.82	2.24	1.42
HBA^e^	5.85	5.18	4.39	3.53
HBD^f^	2.39	1.40	1.29	0.81
NRB^g^	5.31	4.51	3.39	2.06
Log *B/B*^h^	−1.30	−0.77	−0.64	−0.36
BI*P*_Caco-2_ (nm s^−1^)^i^	1,199.37	1,216.27	1,207.91	1,577.16
*S*_mol_ (Å^2^)^j^	696.28	569.69	501.44	393.20
*S*_mol,hfob_ (Å^2^)^k^	409.24	280.66	200.91	131.61
*V*_mol_ (Å^3^)^l^	1,304.41	1,024.64	870.65	645.16
Log*S*_wat_ (*S* in mol L^−1^)^m^	−5.11	−3.87	−3.13	−1.77
Log*K*_HSA_^n^	0.46	0.15	−0.05	−0.44
MDCK^o^	661.25	671.02	663.83	907.31
Ind_coh_^p^	0.013	0.009	0.009	0.006
Glob^q^	0.84	0.86	0.88	0.92
QP_polrz_ (Å^3^)^r^	42.47	33.56	28.23	19.86
LogHERG^s^	−4.64	−4.41	−4.22	−3.40
Log*K*_p_^t^	−2.96	−2.86	−2.89	−2.63
#metab^u^	5.56	4.62	3.57	2.07

^a^Size or number of compounds in library; ^b^number of compounds with #star = 0; ^c^molar weight (range for 95% of drugs is 130 to 725 Da); ^d^logarithm of partitioning coefficient between *n*-octanol and water phases (range for 95% of drugs is −2 to 6); ^e^number of hydrogen bonds accepted by the molecule (range for 95% of drugs is 2 to 20); ^f^number of hydrogen bonds donated by the molecule (range for 95% of drugs is 0 to 6); ^g^number of rotatable bonds (range for 95% of drugs is 0 to 15); ^h^logarithm of predicted blood/brain barrier partition coefficient (range for 95% of drugs is −3.0 to 1.0); ^i^predicted apparent Caco-2 cell membrane permeability in Boehringer-Ingelheim scale, in nm/s (range for 95% of drugs is <5 low, >100 high); ^j^total solvent-accessible molecular surface, in Å^2^ (probe radius 1.4 Å; range for 95% of drugs is 300 to 1,000 Å^2^); ^k^hydrophobic portion of the solvent-accessible molecular surface, in Å^2^ (probe radius 1.4 Å; range for 95% of drugs is 0 to 750 Å^2^); ^l^total volume of molecule enclosed by solvent-accessible molecular surface, in Å^3^ (probe radius 1.4 Å; range for 95% of drugs is 500 to 2,000 Å^3^); ^m^logarithm of aqueous solubility in g/dm^3^ (range for 95% of drugs is −6.0 to 0.5); ^n^logarithm of predicted binding constant to human serum albumin (range for 95% of drugs is −1.5 to 1.2); ^o^predicted apparent MDCK cell permeability in nm/sec (<25 poor, >500 great); ^p^index of cohesion interaction in solids (0.0 to 0.05 for 95% of drugs); ^q^globularity descriptor (0.75 to 0.95 for 95% of drugs); ^r^predicted polarizability (13.0 to 70.0 for 95% of drugs); ^s^predicted IC_50_ value for blockage of HERG K^+^ channels (concern <−5); ^t^predicted skin permeability (−8.0 to −1.0 for 95% of drugs); ^u^number of likely metabolic reactions (range for 95% of drugs is 0 to 15).

**Table 2:** Percentage compliances of selected ADMET-related descriptors of total CamMedNP library in comparison with various subsets

	**Library name**	**Subset**		
	**Total library**	**Drug-like**	**Lead-like**	**Fragment-like**
Log *B/B*	88.22	99.55	100.00	100.00
BI*P*_Caco-2_ (nm s^−1^)	43.95	41.80	39.04	25.93
*S*_mol_ (Å^2^)	89.69	99.55	100.00	95.06
*S*_mol,hfob_ (Å^2^)	90.89	100.00	100.00	100.00
*V*_mol_ (Å^3^)	90.95	99.47	99.81	95.06
Log*S*_wat_ (*S* in mol L^−1^)	69.08	89.57	100.00	97.53
Log*K*_HSA_	85.77	99.82	100.00	100.00
MDCK	49.94	58.02	56.73	49.38
Ind_coh_	95.20	98.75	99.62	100.00
Glob	87.90	96.97	96.73	83.95
ro3^a^	47.22	72.28	91.92	100.00
LogHERG	55.02	61.94	73.27	100.00
Log*K*_p_	91.44	95.99	97.50	97.53
#metab	79.61	89.30	97.31	93.83

The descriptors of the entries in the first column are defined in Table 1; ^a^percentage compliance to Jorgensen's Rule of Three.

### Bioavailability prediction

**Figure 2:**
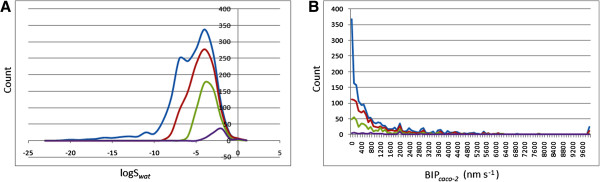
**Distribution curves for compliance to Jorgensen's ‘Rule of Three’. (A)** calculated log*S*_wat_ against count. **(B)** Predicted BI*P*_Caco-2_ against count. Blue = CamMedNP library, red = drug-like subset, green = lead-like subset and violet = fragment-like subset.

**Figure 3:**
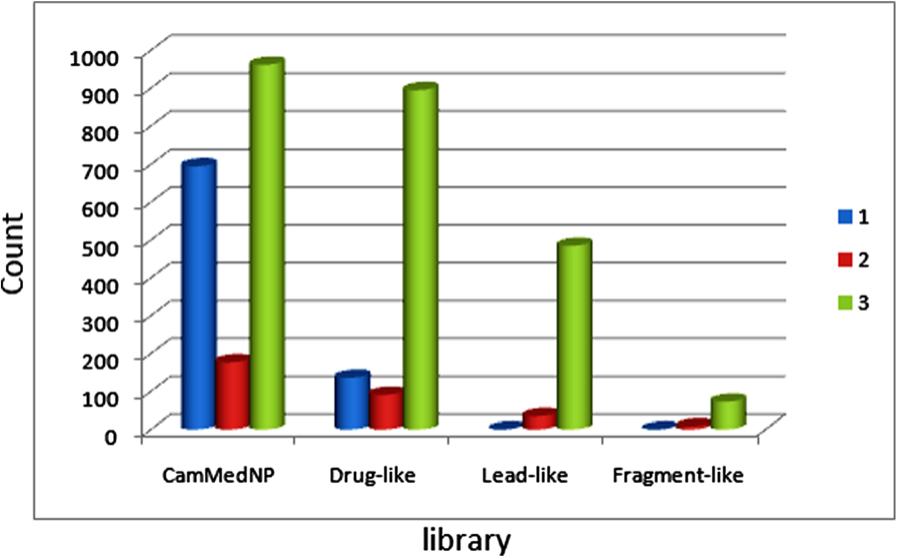
Histograms showing the distribution of human oral absorption predictions.

The bioavailability of a compound depends on the processes of absorption and liver first-pass metabolism [57]. The absorption, in turn, depends on the solubility and permeability of the compound, as well as on the interactions with transporters and metabolizing enzymes in the gut wall. The computed parameters used to assess oral absorption are the predicted aqueous solubility, log*S*_wat_, the conformation-independent predicted aqueous solubility, CI log*S*_wat_, the predicted qualitative human oral absorption, the predicted % human oral absorption and compliance to Jorgensen's famous ‘Rule of Three’ (ro3). The solubility calculation procedure implemented depends on the similarity property space between the given molecule and its most similar analogue within the experimental training set used to develop the model implemented in QikProp, i.e. if the similarity is <0.9, then the QikProp predicted value is taken; otherwise, the predicted property, *P*_pred_, is adjusted such that

(1) $$P_{\mathrm{pred}}=SP_{\exp}+\left(1-S\right)P_{\mathrm{QP}}$$

where *S* is the similarity and *P*_exp_ and *P*_QP_ are, respectively, the experimental and QikProp predictions for the most similar molecule within the training set. In Equation 1, if *S* = 1, then the predicted property is equal to the measured experimental property of the training set compound. According to Jorgensen's ro3, if a compound complies to all or some of the rules (log*S*_wat_ > −5.7, BI*P*_Caco-2_ > 22 nm/s and number of primary metabolites < seven), then it is more likely to be orally available. The distribution curves for two of the three determinants for the ro3 (log*S*_wat_ and BI*P*_Caco-2_) are shown in Figure 2A,B. In general, 47.22% of the CamMedNP library was compliant to the ro3, while the respective percentage compliances for the various subsets were 72.28%, 92.11% and 100% for the drug-like, lead-like and fragment-like libraries. Among the individual computed parameters, the most remarkable was log*S*_wat_, which was met by 75.74% of the compounds within the CamMedNP library, while this property shows a Gaussian distribution for the drug-like and lead-like subsets. Only 37.94% of the compounds fell within the respected range for the BI*P*_Caco-2_ criterion. The predicted apparent Caco-2 cell permeability, BI*P*_Caco-2_ (in nm s^−1^), models the permeability of the gut-blood barrier (for non-active transport), even though this parameter is not often correctly predicted computationally [58]. The histograms of the predicted qualitative human oral absorption parameter (in the scale 1 = low, 2 = medium and 3 = high) are shown in Figure 3. It was observed that 52.45% of the compounds in CamMedNP were predicted to have high human oral absorption. The predicted % human oral absorption (on 0 to 100% scale) shows a similar trend, with 41.06% of the compounds being predicted to be absorbed at 100%, and 57.96% of the compounds predicted to be absorbed at >90%.

The size of a molecule, as well as its capacity to make hydrogen bonds, its overall lipophilicity, its shape and flexibility are important properties to consider when determining permeability. Molecular flexibility has been seen as a parameter which is dependent on the number of rotatable bonds (NRB), a property which influences the bioavailability in rats [58]. The distribution of the NRB for this dataset has been previously discussed [29] and revealed that the compounds within the CamMedNP library show some degree of conformational flexibility, the peak value for the NRB being between 1 and 2, while the average value is 5.31 (Table 1).

### Prediction of blood-brain barrier penetration

**Figure 4:**
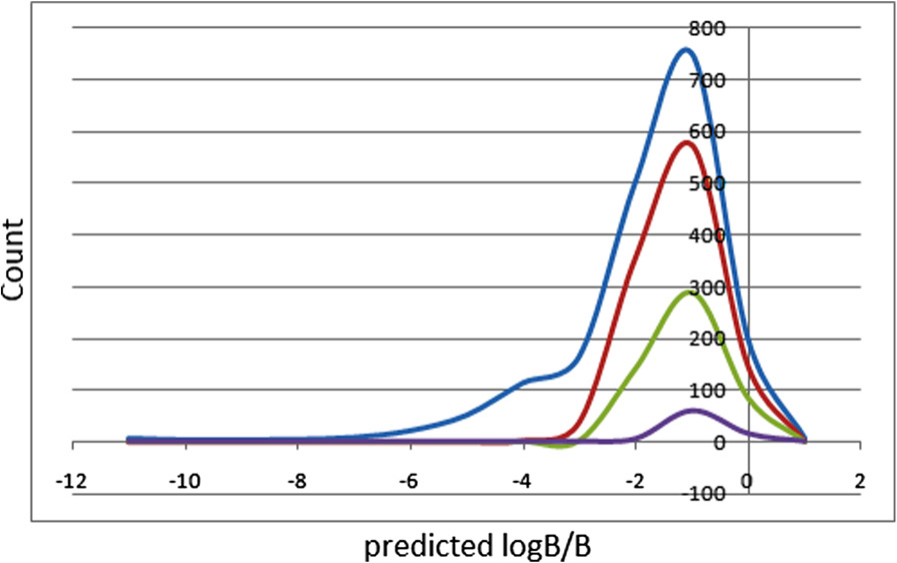
**Plot of the physico-chemical descriptor used to predict BBB penetration.** Predicted log *B/B* against count. The *x*-axis label is the lower limit of the binned data, e.g. 0 is equivalent to 0.0 to 1.0. Blue = CamMedNP library, red = drug-like subset, green = lead-like subset and violet = fragment-like subset.

Too polar drugs do not cross the BBB. The blood/brain partition coefficients (log *B/B*) were computed and used as a predictor for access to the central nervous system (CNS). The predicted CNS activity was computed on a −2 (inactive) to +2 (active) scale and showed that only 1.85% of the compounds in the CamMedNP could be active in the CNS (predicted CNS activity >1). A distribution of the log *B/B* (Figure 4) shows a right-slanted Gaussian-shaped curve with a peak at −0.5 log *B/B* units (the same for all the standard subsets), with >88% of the compounds in the CamMedNP falling within the recommended range for the predicted brain/blood partition coefficient (−3.0 to 1.2). The MDCK monolayers are widely used to make oral absorption estimates, the reason being that these cells also express transporter proteins, but only express very low levels of metabolizing enzymes [58]. They are also used as an additional criterion to predict BBB penetration. Thus, our calculated apparent MDCK cell permeability could be considered to be a good mimic for the BBB (for non-active transport). It was estimated that only about 50% of the compounds had apparent MDCK cell permeabilities falling within the recommended range of 25 to 500 nm s^−1^ for 95% of known drugs. This situation was not greatly improved in the drug-like and lead-like subsets (58% and 57%, respectively).

### Prediction of dermal penetration

**Figure 5:**
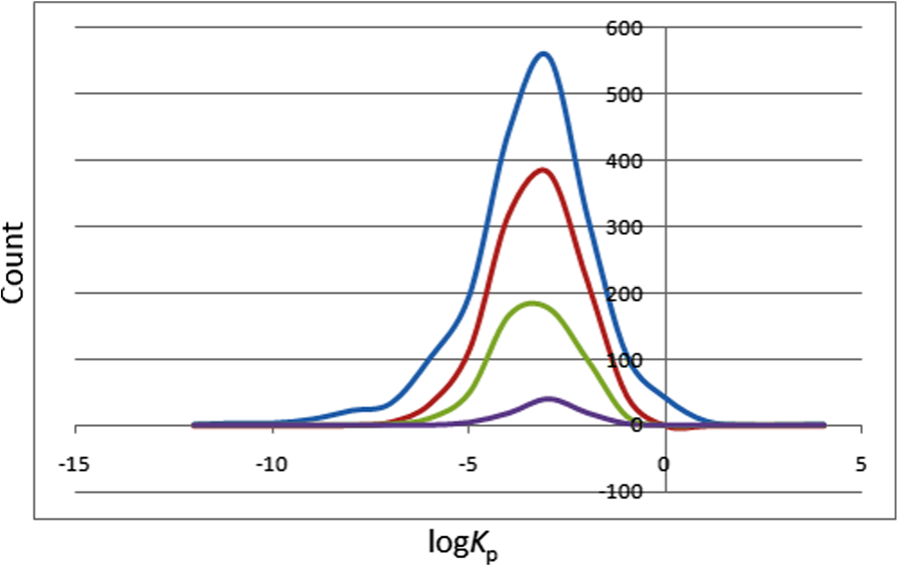
**Distribution curves for the predicted skin penetration parameter.** Blue = CamMedNP library, red = drug-like subset, green = lead-like subset and violet = fragment-like subset.

This factor is important for drugs administered through the skin. The distribution of computed skin permeability parameter, log*K*_p_, showed smooth Gaussian-shaped graphs centred at −2.5 log*K*_p_ units for all the four datasets (Figure 5), with approximately 91% of the compounds in the CamMedNP database falling within the recommended range for >95% of known drugs. The predicted maximum transdermal transport rates, *J*_m_ (in μ cm^−2^ h^−1^), were computed from the aqueous solubility (*S*_wat_), the MW and skin permeability (*K*_p_) using the relation (2):

(2) $$J_{m}=K_{p}\times \mathrm{MW}\times S_{\mathrm{wat}}$$

This parameter showed variations from 0 to 1,603 μ cm^−2^ h^−1^, with only about 1.39% of the compounds in CamMedNP having the predicted value of *J*_m_ > 100 μ cm^−2^ h^−1^.

### Prediction of plasma-protein binding

**Figure 6:**
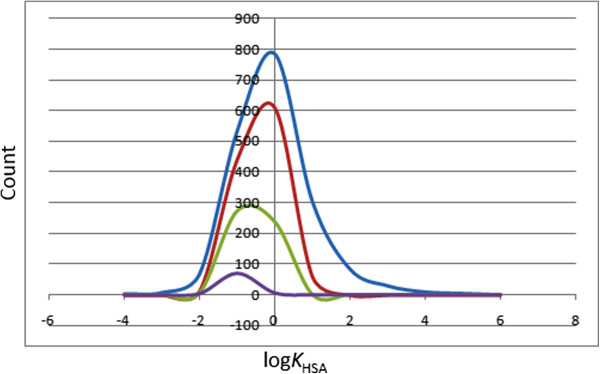
**Distribution curves for predicted plasma-protein binding.** Blue = CamMedNP library, red = drug-like subset, green = lead-like subset and violet = fragment-like subset.

The efficiency of a drug may be affected by the degree to which it binds to the proteins within the blood plasma. It is noteworthy that the binding of drugs to the plasma proteins (like human serum albumin, lipoprotein, glycoprotein, α, β and γ globulins) greatly reduces the quantity of the drug in the general blood circulation, and hence, the less bound a drug is, the more efficiently it can traverse cell membranes or diffuse. The predicted plasma-protein binding has been estimated by the prediction of binding to human serum albumin; the log*K*_HSA_ parameter recommended range is −1.5 to 1.5 for 95% of known drugs. Figure 6 shows the variation of this calculated parameter within the CamMedNP dataset, as well as for the standard subsets. This equally gave smooth Gaussian-shaped curves centred on −0.5 log*K*_HSA_ units for all the four datasets. In addition, our calculations revealed that >85% of the compounds within the CamMedNP library are compliant to this parameter, indicating that a majority of the compounds are likely to circulate freely within the blood stream and, hence, have access to the target site.

### Metabolism prediction

**Figure 7:**
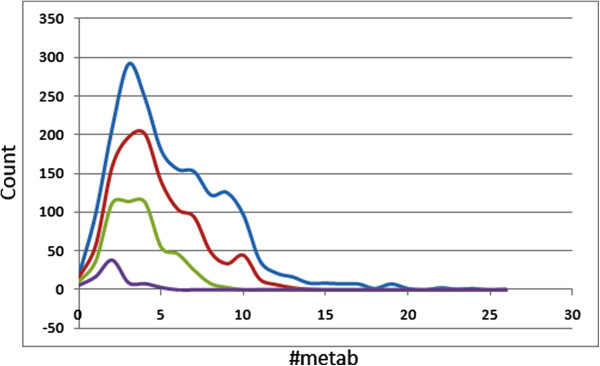
**Distribution of the predicted number of metabolic reactions for compounds in the CamMedNP.** Blue = CamMedNP library, red = drug-like subset, green = lead-like subset and violet = fragment-like subset.

An estimated number of possible metabolic reactions has also been predicted by QikProp and used to determine whether the molecules can easily gain access to the target site after entering the blood stream. The average estimated number of possible metabolic reactions for the CamMedNP library was between five and six, while those of the standard subsets drop sequentially by one step in a progressive manner (Table 1). Even though some of the compounds are likely to undergo as many as up to 26 metabolic reactions due to the complexity of some of the plant secondary metabolites within the database (Figure 7), about 80% of the compounds are predicted to undergo the recommended number of metabolic steps (one to eight reactions), with the situation improving to around 90% and approximately 97% in the drug-like and lead-like subsets, respectively. From Figure 7, it can be observed that, except for the fragment-like subsets which peaks at two predicted metabolic reactions, the peak values for the number of predicted metabolic reactions were at three for all of the datasets.

### Prediction of blockage of human ether-a-go-go-related gene potassium channel

**Figure 8:**
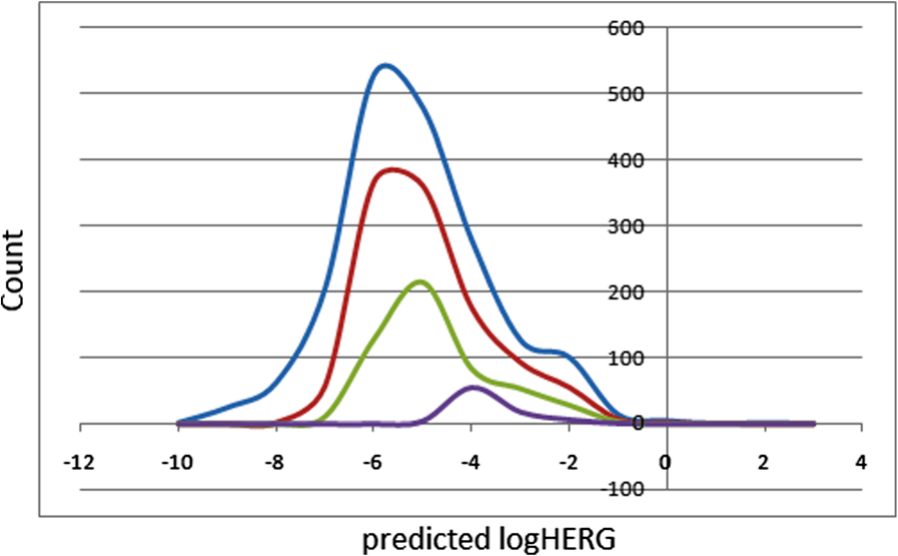
**A plot of the predicted logHERG values for the CamMedNP and standard subsets.** Blue = CamMedNP library, red = drug-like subset, green = lead-like subset and violet = fragment-like subset.

Human ether-a-go-go-related gene (HERG) encodes a potassium ion (K^+^) channel that is implicated in the fatal arrhythmia known as *torsade de pointes* or the long QT syndrome [59]. The HERG K^+^ channel, which is best known for its contribution to the electrical activity of the heart which coordinates the heart's beating, appears to be the molecular target responsible for the cardiac toxicity of a wide range of therapeutic drugs [60]. HERG has also been associated with modulating the functions of some cells of the nervous system and with establishing and maintaining cancer-like features in leukemic cells [61]. Thus, HERG K^+^ channel blockers are potentially toxic, and the predicted IC_50_ values often provide reasonable predictions for cardiac toxicity of drugs in the early stages of drug discovery [62]. In this work, the estimated or predicted IC_50_ values for blockage of this channel have been used to model the process *in silico*. The recommended range for the predicted log IC_50_ values for blockage of the HERG K^+^ channels (logHERG) is >−5. A distribution curve for the variation of the predicted logHERG is shown in Figure 8, which is left-slanted Gaussian-shaped curve with a peak at −5.5 logHERG units for both the total library and the drug-like subset, meanwhile the lead-like library rather peaks at −4.5 units. It was observed that, in general, this parameter is predicted to fall within the recommended range for about 55% of the compounds within the CamMedNP database, approximately 62% for the drug-like subset and around 73% for the lead-like subset.

### Usefulness of the CamMedNP library

The usefulness of the CamMedNP database in lead generation has been exemplified with the docking and pharmacophore-based screening for potential inhibitors of a validated anti-malarial drug target in our laboratory, and the results will be published in a subsequent paper. It is important to mention that virtual screening results could provide insight and direct natural products chemists to search for theoretically active principles with attractive ADMET profiles, which have been previously isolated, but not tested for activity against specified drug targets (if samples are absent). This ‘resurrection’ process could prove to be a better procedure for lead search than the random screening, which is a common practice in our Cameroonian laboratories. CamMedNP is constantly being updated; meanwhile, a MySQL platform (Cupertino, USA) to facilitate the searching of this database and ordering of compound samples is under development within our group and will also be published subsequently. However, 3D structures of the compounds, as well as their physico-chemical properties that were used to evaluate the DMPK profile, can be freely downloaded as additional files accompanying this publication (see Additional file 1, Additional file 2, Additional file 3, Additional file 4). In addition, information about compound sample availability can be obtained on request from the authors of this paper or from the pan-African Natural Products Library (p-ANAPL) project [63,64].

## Conclusion

Modern drug discovery programs usually involve the search for small molecule leads with attractive pharmacokinetic profiles. The presence of such within the CamMedNP library is of major importance and, therefore, renders the database attractive, in addition to the already-known properties (drug-like, lead-like fragment-like and diverse). This is an indication that the 3D structures of naturally occurring compounds within the CamMedNP could be a good starting point for docking, neural networking and pharmacophore-based virtual screening campaigns, thus rendering the CamMedNP as a useful asset for the drug discovery community. 3D structures of the compounds, as well as their physico-chemical properties that were used to evaluate the DMPK profile of the CamMedNP library, can be freely downloaded (for non-commercial use) as additional files which accompany this publication (see Additional file 1, Additional file 2, Additional file 3, Additional file 4). The physical samples for testing are available at the various research laboratories in Cameroon in varying quantities. Questions regarding the availability of the compound samples could be addressed directly to the authors of this paper. Otherwise, the samples could be obtainable from the p-ANAPL consortium, which has a mandate to collect samples of NPs from the entire continent of Africa and make them available for biological screening. This network is being set up under the auspices of the Network for Analytical and Bioassay Services in Africa [63,64].

## Abbreviations

3D: Three dimensional; ADME/T: Absorption distribution, metabolism, excretion and toxicity; CamMedNP: Cameroonian medicinal plant and natural products database; DMPK: Drug metabolism and pharmacokinetics; log P: logarithm of the octan-1-ol/water partition coefficient; MDCK: Madin-Darby canine kidney; MW: Molar weight; NP: Natural product; NRB: Number of rotatable bonds; p-ANAPL: pan-African natural products library

## Competing interests

The authors declare that they have no competing interests.

## Authors' contributions

WS, EM, LCOO and SMNE conceived the project. All authors participated in the data generation and analysis, discussion of results and the conception of the paper. FNK wrote the first draft of the paper. This work is part of the PhD project of FNK. All authors read and approved the final manuscript.

## Authors' information

WS and SMNE are professors of Medicinal Chemistry with an interest in CADD, while SMNE also focuses on organic synthesis and on natural product leads from the Cameroonian medicinal plants. LMM and JAM are natural products chemists actively involved in the isolation and characterization of secondary metabolites from the Cameroonian medicinal plants. LLL holds a PhD in Environmental Science and manages the Chemical and Bioactivity Information Centre (CBIC) with a focus on developing databases for information from medicinal herbs in Africa. PNJ is a retired research officer of Lhasa Ltd., who currently leads the CBIC branch in Leeds, UK. FNK is a PhD student working on CADD under the joint supervision of LCOO and EM.

## Supplementary Material

Additional file 1 **Compounds currently included in CamMedNP.** 1 3D structures of compounds currently included in CamMedNP with calculated pharmacokinetic descriptors. Click here for file

Additional file 2 **Drug-like subset.** 3D structures of the drug-like subset with calculated pharmacokinetic descriptors. Click here for file

Additional file 3 **Lead-like subset.** 3D structures of the lead-like subset with calculated pharmacokinetic descriptors. Click here for file

Additional file 4 **Fragment-like subset.** 3D structures of the fragment-like subset with calculated pharmacokinetic descriptors. Click here for file
